# Health-related quality of life among breast cancer patients compared to cancer survivors and age-matched women in the general population in Vietnam

**DOI:** 10.1007/s11136-021-02997-w

**Published:** 2021-09-20

**Authors:** Tran Thu Ngan, Vu Quynh Mai, Hoang Van Minh, Michael Donnelly, Ciaran O’Neill

**Affiliations:** 1 Centre for Public Health, Queen’s University Belfast, Belfast, UK; 2 Centre for Population Health Sciences, Hanoi University of Public Health, Hanoi, Vietnam; 3 Department of Epidemiology and Global Health, Umea University, Umea, Sweden

**Keywords:** HRQoL, Breast cancer, Health utility, Vietnam, EQ-5D-5L

## Abstract

**Purpose:**

This study compared the health-related quality of life (HRQoL) of breast cancer (BC) patients, survivors, and age-matched women from the general population in Vietnam to address the paucity of HRQoL research and contribute to the robust assessment of BC screening and care in Vietnam.

**Methods:**

The standardised EQ-5D-5L instrument was incorporated in an online survey and a hospital-based face-to-face survey, and together with data from the Vietnam EQ-5D-5L norms study. *χ*^2^ tests assessed EQ-5D health profile associations and a Tobit regression model investigated the association between overall health status (EQ-VAS/utility scores) and sociodemographic and clinical characteristics.

**Results:**

A total of 309 participants (107 patients undergoing treatment and 202 survivors who had completed treatment) provided usable responses. The dimensions that affected mostly the HRQoL of women with BC were pain/discomfort and anxiety/depression. Current patients and survivors differed significantly regarding HRQoL dimensions of mobility, self-care, usual activities, and anxiety/depression. Their health utilities were 0.74 and 0.84, respectively, compared with 0.91 for age-matched Vietnamese women in the general population (*p* < 0.001). Treatment status (survivor vs patient), younger age, higher monthly household income, and higher education levels were associated with higher health utility.

**Conclusions:**

The results point to unmet needs in mental health support and well-being and for attention to be given to the development of a biopsychosocial system of cancer diagnosis, treatment, and care. The results will also inform future assessments of the comparative value for money of interventions intended to impact on breast cancer in Vietnam.

**Supplementary Information:**

The online version contains supplementary material available at 10.1007/s11136-021-02997-w.

## Introduction

Breast cancer (BC) is the most common cancer among Vietnamese women [1]. In 2018, the estimated age-standardized incidence rate for breast cancer in Vietnam was 26.4/100000 which accounted for 20.6% of all new cancer cases in women [1]. This rate is likely to be an underestimate due to the quality and coverage of data from cancer registries [2]. Data from a pilot screening programme of 142000 women in 2013 reported an incidence rate of 58.5/100000 [3]. Nevertheless, the incidence has risen steadily over years [4].

The five-year survival rate of BC patients treated at National Cancer Hospital and Hue Central Hospital was 86.4 and 74%, respectively [5, 6]. The former rate was on a par with several high-income countries such as Japan, Korea, and Canada [6]. However, an assessment of the value of BC tertiary care and other services in Vietnam is seriously hampered by the paucity of health-related quality of life data on BC patients and survivors relative to the general population. Studies reported that around 28% of Vietnamese cancer patients suffered serious depression and/or anxiety [7, 8]. Qualitative research also revealed the problems of overcrowded hospitals and poor communication from health care providers during diagnosis and treatment [9]. Therefore, the need for patient-reported outcomes (PROMs) such as health-related quality of life (HRQoL) is clear, especially to inform cost-effectiveness analyses.

There are very few studies about the HRQoL of BC patients in Vietnam [10, 11] and no studies about the HRQoL of BC survivors. Given the high survival rate and the fact that 64.7% of new BC cases in Vietnam were in women below the age 50 [12], the population of BC survivors is considerable and susceptible to greater potential years of life lost than in Europe or North America. Studies of their HRQoL, therefore, are highly relevant and understanding the HRQoL of BC patients and survivors may provide valuable insights that could help improve treatment and follow-up care as well as contributing to the assessment of novel interventions in terms of value for money [13]. There is a need to address these knowledge gaps in HRQoL including assessing BC burden by comparing (for the first time) the HRQoL of BC patients/survivors and women in the general population. We used the generic instrument, EQ-5D-5L, to assess and compare the HRQoL of BC patients, BC survivors, and age-matched women from the general Vietnamese population.

## Materials and methods

### Study design

We conducted an online survey and a hospital-based survey to reach BC patients and survivors in different geographical locations in Vietnam. Respondents, respectively, either accessed and completed the self-administrative web-based questionnaire or were approached and interviewed face-to-face. The hospital-based survey was conducted at Hanoi Oncology Hospital and Oncology Center of Hue Central Hospital (tertiary cancer treatment hospitals for the Northern and Central regions of the country, respectively). The EQ-5D-5L instrument developed by the EuroQol Group [14] was used to measure HRQoL of BC patients/survivors. The EQ-5D-5L was chosen because of its wide use internationally [15] which enhanced the opportunities to compare results with other studies.

We then used raw data^1^ of an EQ-5D-5L standardised valuation study [16] conducted in 2017 with a nationally representative sample of Vietnamese general population to match each BC patient/survivor (as case) with a peer (woman in general population) by age (as control) (1-to-1 match). Firstly, data were randomly sorted, each case was matched with a ‘nearest’ control with same age or difference within a calliper of 0.25*standard deviation of age (recommended calliper by literature [17]). The matched pair of case and control was removed from the pool before next matching was performed. In short, the nearest neighbour matching within a calliper and nonreplacement was the matching algorithm [17]. After matching, HRQoL was compared amongst groups of BC patients, survivors, and age-matched peers.

### Participants and recruitment

Both the online and hospital-based surveys targeted: (1) BC patients (who were receiving hospital treatment) and (2) BC survivors (who finished treatment and discharged from hospital). Patients who were undergoing investigation for suspected BC but had not received a diagnosis were excluded. BC patients/survivors for the online survey were recruited through official websites and/or social media of national BC organizations as well as (breast) cancer patient/survivor clubs. In addition, all BC patients/survivors who presented at the named tertiary treatment centres whilst the survey was underway were approached and asked to participate. Bearing in mind available resources and logistics of conducting face-to-face interviews, data collection were restricted to three months for the online survey and three days for the hospital surveys. The sample size comprised the number of people who responded by the end of the time restriction.

### Patient involvement

The leaders (who are cancer survivors) of the two biggest BC and cancer (in general) patient/survivor ‘clubs’ in the country were invited to the advisory board of the study. They were not involved in the questionnaire development as the EQ-5D-5L is a standard instrument and its license forbids any changes though they were asked to assess the sensitivity of the questions and whether the time required to answer the questionnaire may cause any burden to the patients during their treatment. The leaders actively contributed to the recruitment process by distributing information related to the study and encouraging their peers to participate. We intend to disseminate the main results to the participants of the study and the general population of BC patients and survivors in Vietnam. The patient leaders will be consulted to choose an appropriate and effective method of dissemination.

### HRQoL variables and measurements

#### Dependent variables: EQ-5D health profile, perceived rating of overall health status (EQ-VAS score), and utility score

Health profile was assessed through respondents’ reported levels of problems in the EQ-5D-5L dimensions: ‘mobility’, ‘self-care’, ‘usual activities’, ‘pain/discomfort’, and ‘anxiety/depression’. Respondents indicated their health state on each dimension by choosing the most appropriate response from: ‘no problems’, ‘slight problems’, ‘moderate problems’, ‘severe problems’, and ‘unable to/extreme problems’.

Respondents also assessed their own overall health status by indicating a score on a visual analogue scale (EQ-VAS score) with values from 0 to 100 corresponding to ‘worst imaginable health’ and ‘best imaginable health’, respectively.

A utility score was derived for each respondent by converting their health profile using the value set of EQ-5D-5L for the general population of Vietnam [16] that reflected the relative weight that Vietnamese adults placed on the problem levels of each health dimension (see Supplementary file 1 for details about the calculation).

#### Independent variable and co-variates

The main independent variable was treatment status (patients vs survivors) derived from the question, “Have you finished treatment and been discharged from hospital?” Co-variates included ‘age’, ‘education’, ‘marital status’, ‘occupation’, ‘residence area’, ‘household monthly income’, ‘cancer stage at diagnosis’, and ‘stage of treatment’ (based on the most recently used health services related to BC). A previous study about HRQoL of BC patients in Vietnam (using a different measurement tool), a systematic review of the HRQoL of Asian BC patients and several similar studies in neighbouring countries [10, 18–20] were used to select covariates and help guide analysis.

### Statistical analysis

Sociodemographic characteristics of BC patients and survivors were compared using t-tests and *χ*^2^ tests. (1) The EQ-5D health profile; (2) The overall self-rated health status EQ-VAS; and (3) The utility scores (EQ-5D values) were used to compare HRQoL among BC patients, survivors, and age-matched women in the general population.

Regarding EQ-5D health profile, the frequencies and proportions of BC patients and survivors reporting each level of problem on each dimension was presented and *χ*^2^ tests were used to assess between-group differences. The level of problems on each dimension were dichotomised into ‘no problems’ and ‘any problems’ to undertake comparisons with age-matched women from the general population.

Corresponding to EQ-VAS and utility scores, descriptive statistics used mean value and the standard deviation (SD). Kruskal Wallis tests assessed differences in BC patients’ median EQ-VAS/utility scores by stage of treatment. The most common regression techniques for analysing EQ-5D data, ordinary least squares (OLS), Tobit, and generalized linear model (GLM) were considered [21] in relation to conducting an assessment of the determinants of EQ-VAS and utility scores. Results from these models were similar. We decided to report the results from Tobit model as it takes account of the censored nature of EQ-5D data (bounds at full health and worst health state) [21]. Results from OLS and GLM models are provided in the Supplementary file 2. Two Tobit-derived models were presented. Model 1 contains all covariates based on a literature review and binary analyses including treatment status (patients vs survivors), age, education level, residence area (urban vs rural), marital status, and household monthly income. Model 2 consisted only of those independent variables that were statistically significantly associated with EQ-VAS/utility scores (identified via a backward elimination approach). Model goodness-of-fit was compared using Akaike and Bayesian information criteria (AIC and BIC).

## Results

### Characteristics of study participants

**Fig. 1:**
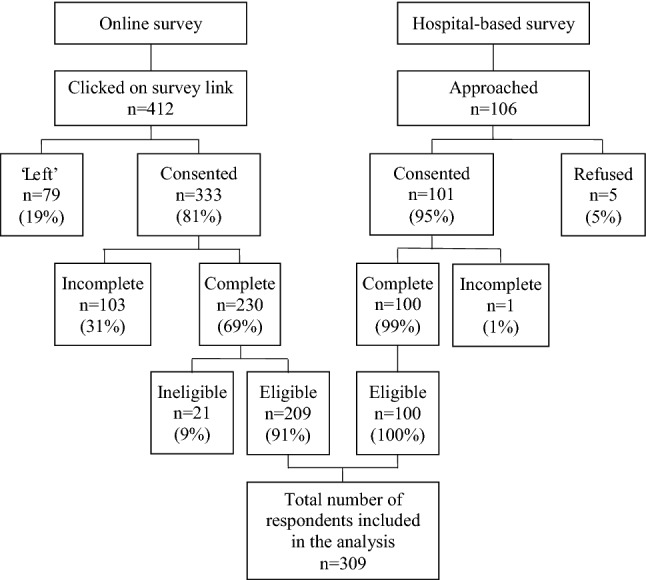
Recruitment process and results

The online survey was promoted via the communication channels of eight different stakeholders and remained open from September 3 to December 3, 2019. During this period, 412 individuals clicked the survey link, 333 (81%) consented, and 230 (69%) completed the questionnaire; 21 were removed because they were non-breast cancer patients/survivors, leaving 209 respondents. In the hospital-based survey, 106 BC patients/survivors were approached, 101 (95%) consented, and 100 (99%) completed interviews. Thus, the combined dataset of breast cancer patients/survivors consisted of 309 observations (Fig. 1).

Table 1 presents the sociodemographic characteristics of participants. There were no significant differences in terms of age, marital status, and occupation between BC patients and survivors. A significantly greater proportion of survivors attained a higher education level/monthly income and lived in urban areas. Compared with national data [22–24], the combined study sample contained a higher proportion of individuals who lived in urban areas and spent more years in formal education.

**Table 1:** Sociodemographic characteristics of participants

Characteristics	BC Patients	BC Survivors	*P*-value	Total	National data
	*n* (%)	*n* (%)		*n* (%)	*n* (%)
Total	107 (34.6)	202 (65.4)		309 (100.0)	
Age, mean (SD)	47 (11)	48 (10)	NS	48 (10)	NA
Education level					
No formal education/not completed primary education	9 (8.5)	6 (3.0)	< 0.001	15 (4.9)	79.2^a^
Having completed primary education	11 (10.4)	5 (2.5)		16 (5.2)	
Having completed secondary education	23 (21.7)	16 (8.0)		39 (12.7)	
Having completed high school education	19 (17.9)	38 (18.9)		57 (18.6)	20.8^a^
Graduated university/college/vocational education	38 (35.8)	122 (60.7)		160 (52.1)	
Having completed post-graduate	6 (5.7)	14 (7.0)		20 (6.5)	
Marital status					
Single/separated/divorced/widow	26 (24.8)	44 (22.0)	NS	70 (23.0)	NA
Married	79 (75.2)	156 (78.0)		235 (77.0)	
Occupation					
Government employee	25 (23.6)	64 (32.2)	NS	89 (29.2)	45.0^b^
Non-government employee	12 (11.3)	32 (16.1)		44 (14.4)	
Self-employed (included subsistence farming)	35 (33.0)	44 (22.1)		79 (25.9)	NA
Student/Homemaker/Housewife	11 (10.4)	16 (8.0)		27 (8.8)	
Retired	15 (14.2)	36 (18.1)		51 (16.7)	
Unemployed	8 (7.5)	7 (3.5)		15 (4.9)	
Residence area: urban	51 (53.7)	157 (77.7)	< 0.001	211 (70.1)	35.0^c^
Household monthly income					
≤ 3000000 VND (~ £100)	25 (24.5)	18 (9.2)	0.001	43 (14.5)	NA
3000001–6000000 VND (~ £100–200)	24 (23.5)	33 (16.9)		57 (19.2)	
6000001–9000000 VND (~ £200–300)	11 (10.8)	20 (10.3)		31 (10.4)	
9000001–12000000 VND (~ £300–400)	20 (19.6)	55 (28.2)		75 (25.3)	
> 12000000 VND (~ £400)	22 (21.6)	69 (35.4)		91 (30.6)	

Exchange rate in October 2020: £1 ~ 30,000 VND

*BC* breast cancer, *NA* not applicable/available, *NS* not significant, *SD* standard deviation

*VND* vietnamese dong

^a^Data are from Vietnam population and housing census 2009 [22]

^b^Data are from 2018 report of the ministry of labour—invalids and social affairs [23]

^c^Data are from Statistical Summary Book of Vietnam 2017 [24]

### Health profile across five health dimensions

Regarding mobility, self-care, usual activities, and anxiety/depression, a significantly greater proportion of current patients reported problems compared to survivors (Table 2). Pain/discomfort was the only dimension for which there was no statistically significant difference between patients and survivors. Overall, pain/discomfort and anxiety/depression were the two dimensions in which the highest proportion of respondents reported having any problems (75 and 57%, respectively). Self-care was the least affected dimension—only 17.6% respondents reported problems in this dimension.

**Table 2:** EQ-5D-5L frequencies and proportions reported by dimension and level

	Total *n* (%)	BC patients *n* (%)	BC survivors *n* (%)	*p*-value*
Mobility				
No problems	221 (71.5)	65 (60.7)	156 (77.2)	0.014
Slight problems	65 (21.0)	30 (28.0)	35 (17.3)	
Moderate problems	12 (3.9)	8 (7.5)	4 (2.0)	
Severe problems	10 (3.2)	4 (3.7)	6 (3.0)	
Unable/extreme problems	1 (0.3)	0 (0.0)	1 (0.5)	
Self-care				
No problems	255 (82.5)	74 (69.2)	181 (89.6)	< 0.001
Slight problems	36 (11.7)	18 (16.8)	18 (8.9)	
Moderate problems	11 (3.6)	9 (8.4)	2 (1.0)	
Severe problems	7 (2.3)	6 (5.6)	1 (0.5)	
Usual activities				
No problems	209 (67.6)	64 (59.8)	145 (71.8)	0.002
Slight problems	83 (26.9)	30 (28.0)	53 (26.2)	
Moderate problems	10 (3.2)	8 (7.5)	2 (1.0)	
Severe problems	6 (1.9)	5 (4.7)	1 (0.5)	
Unable/extreme problems	1 (0.3)	0 (0.0)	1 (0.5)	
Pain/discomfort				
No problems	76 (24.6)	22 (20.6)	54 (26.7)	NS
Slight problems	170 (55.0)	54 (50.5)	116 (57.4)	
Moderate problems	40 (12.9)	19 (17.8)	21 (10.4)	
Severe problems	19 (6.1)	8 (7.5)	11 (5.4)	
Unable/extreme problems	4 (1.3)	4 (3.7)	0 (0.0)	
Anxiety/depression				
No problems	132 (42.7)	32 (29.9)	100 (49.5)	< 0.001
Slight problems	116 (37.5)	37 (34.6)	79 (39.1)	
Moderate problems	27 (8.7)	14 (13.1)	13 (6.4)	
Severe problems	26 (8.4)	17 (15.9)	9 (4.5)	
Unable/extreme problems	8 (2.6)	7 (6.5)	1 (0.5)	

*BC* breast cancer, *NS* not significant

*Results of *χ*^2^ tests compared between groups of patients and survivors (this was before matching with peers from the general population)

**Fig. 2:**
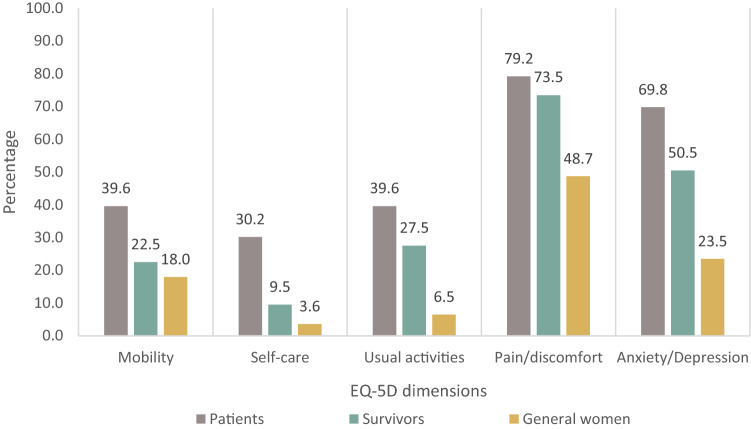
Proportion of respondents who reported problems on each EQ-5D dimension

306/309 BC patients/survivors were age-matched with their peers in the general population using the raw dataset of the EQ-5D standardised valuation study [16] conducted in 2017 in Vietnam (which consisted of a national representative sample of 613 women aged 18 +). Figure 2 shows the results regarding EQ-5D health profile after the matching. A significantly greater proportion of BC patients/survivors reported problems in all five EQ-5D dimensions (Chi-square tests, *p* < 0.001, test results are not shown in the figure) compared to women from the general population. A clear and consistent group hierarchy is evident in terms of experience of problems across domains.

### EQ-VAS and utility scores

**Fig. 3:**
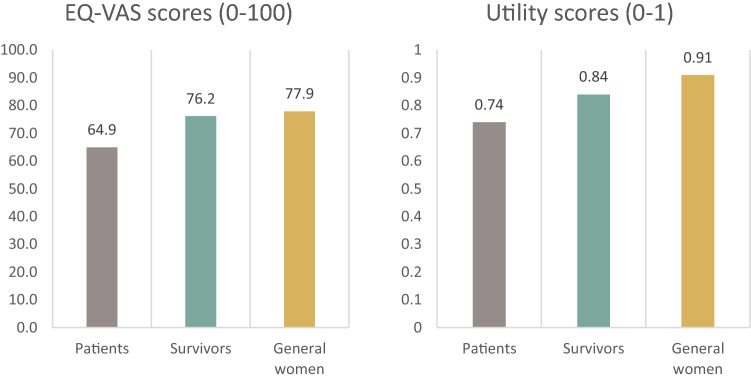
EQ-VAS/utility scores for breast cancer patients, survivors, and age-matched women from the general population

The mean (SD) EQ-VAS and utility scores of BC patients were 64.9 (20.1) and 0.74 (0.22) which were significantly lower than BC survivors, 76.2 (15.7) and 0.84 (0.15), respectively (Mann Whitney *U* test, Z = − 4.2, *p* < 0.001) (Fig. 3, test results are not shown). These scores were lower than age-matched women from the general population, 77.9 (15.0) and 0.91 (0.1), respectively.

Table 3 shows BC patients’ mean EQ-VAS and utility scores by stage of treatment. Although EQ-VAS scores and utility scores showed clear differences among those at different stages of treatment, the difference was not statistically significant (Kruskal–Wallis tests). Lowest scores were reported when the patients have just finished mastectomy (mean EQ-VAS score = 54, mean utility score = 0.68). The next stages with low scores included chemotherapy, radiotherapy, and targeted therapy with scores ranged from 61.8 to 70.1 for EQ-VAS and 0.71 to 0.80 for utility. In most cases, scores of those in active treatment were lower than those of the survivors except in the cases of lumpectomy and breast reconstruction surgery where patients had higher EQ-VAS and utility scores than survivors (EQ-VAS: 90.5 and 80.0 vs 76.2; Utility score: 0.88 and 0.92 vs 0.84, respectively).

**Table 3:** Breast cancer patients’ mean EQ-VAS/utility scores by stage of treatment

	EQ-VAS scores			Utility scores		
	*n*	mean (SD)	*p*-value**	*n*	mean (SD)	*p*-value**
Patients’ stage of treatment*						
Lumpectomy	2	90.5 (0.7)	NS	2	0.88 (0.08)	NS
Mastectomy	10	54.0 (29.4)		10	0.68 (0.30)	
Breast reconstruction surgery	1	80.0 (0.0)		1	0.92 (0.00)	
Chemotherapy	51	61.8 (19.2)		51	0.71 (0.24)	
Targeted (biological) therapy	14	69.6 (15.6)		14	0.80 (0.13)	
Radiotherapy	24	70.1 (21.7)		24	0.77 (0.20)	
Total	102	64.8 (20.4)		102	0.74 (0.22)	

*NS* not significant, *SD* standard deviation

*Patients’ stage of treatment was defined based on the current (if patients were undergoing a treatment) or most recent (if patients had just finished one treatment therapy and were waiting for the next one) use of health services related to breast cancer treatment. This was excluded newly diagnosed patients who had not yet received any treatment

**Difference among stage of treatment groups was analysed with the Kruskal–Wallis tests

In the Tobit models, compared with BC patients, the survivors reported a 9.3 point higher score EQ-VAS (*β* = 9.5, *p* < 0.001; Model 1: treatment status was adjusted for age group, education level, household monthly income, residence area, and marital status) and 0.07 higher utility score (*β* = 0.07, *p* < 0.001; Model 2: treatment status was adjusted for age group, education level, and household monthly income) (Table 4).

**Table 4:** Tobit model analyses of EQ-5D-5L utility scores and EQ-VAS scores

Variable	EQ-VAS score				Utility score			
	Model 1^a^ *n* = 287		Model 2^b^ *n* = 306		Model 1^a^ *n* = 288		Model 2^b^ *n* = 296	
	*β* coeff	95% CI	*β* coeff	95% CI	*β* coeff	95% CI	*β* coeff	95% CI
Treatment status								
Patient^ref^								
Survivor	9.3*	**4.5–14.1**	9.0*	**4.4–13.6**	0.06*	**0.01–0.11**	0.07*	**0.02–0.11**
Age group, years								
< 40^ref^								
40–49	− 0.1	− 5.1–4.9	− 0.6	− 5.3–4.1	− 0.05	− 0.09–0.00	− 0.04	− 0.08–0.00
50–59	− 4.5	− 10.7–1.6	− 4.8	− 10.6–0.9	− 0.04	− 0.12–0.03	− 0.04	− 0.09–0.02
60 +	− 9.4*	**− 16.9–− 1.8**	− 9.2*	**− 16.2–− 2.2**	− 0.11*	**− 0.18–− 0.03**	− 0.10*	**− 0.17–− 0.03**
Residence								
Rural^ref^								
Urban	1.1	− 4.2–6.5			0.03	− 0.04–0.10		
Education level								
Completed up to secondary school^ref^								
Completed high school	4.7	− 3.2–12.6	5.8	− 1.1–12.8	0.05	− 0.03–0.13	0.06	− 0.02–0.15
Completed graduate	5.8	− 1.1–12.7	7.8*	**2.2–13.4**	0.06	− 0.01–0.13	0.08*	**0.01–0.15**
Completed postgraduate	7.4	− 3.8–18.6	9.6*	**0.5–18.7**	0.10*	**0.00–0.19**	0.12*	**0.03–0.21**
Marital status								
Single/separated/divorce/widow^ref^								
Married	− 1.1	− 6.5–4.3			0.01	− 0.01–0.09		
Household monthly income								
≤ 3000000 VND (~ £100)^ref^								
3000001–6000000 VND (~ £100–200)	2.0	− 6.5–10.4			0.04	− 0.06–0.14	0.04	− 0.05–0.14
6000001–9000000 VND (~ £200–300)	3.9	− 5.0–12.7			0.07	− 0.03–0.17	0.07	− 0.02–0.17
9000001–12000000 VND (~ £300–400)	2.4	− 6.2–11.0			0.09	− 0.01–0.18	0.09*	**0.00–0.18**
> 12000000 VND (~ £400)	3.0	− 5.6–11.5			0.07	− 0.02–0.16	0.08	–0.01–0.17
AIC	**2463**		**2605**		**− 176.5**		**− 191.6**	
BIC	**2518**		**2638**		**− 121.5**		**− 143.7**	

Exchange rate in October 2020: £1 ~ 30,000 VND

*β coeff* beta coefficients of the Tobit model, ^*ref*^ reference group, *AIC* akaike information criteria, *BIC* bayesian information criteria, *VND* vietnamese dong

*p < 0.05 versus reference group

^a^Model 1 with all exploratory variables

^b^Model 2 included only variables with significant coefficients (Backward elimination). Both model 1 and 2 included only complete cases (observations with missing data were excluded) lead to the total number of observations in each model (*n*) was different

Sociodemographic characteristics significantly associated with both EQ-VAS and utility scores were age, education, and income level. Specifically, from the age 60, advancing age was significantly associated with a negative impact on both EQ-VAS scores and utility scores (this trend was not presented in the group under 60 years old). Completion of university or above and having household monthly income in the range of 9000001–12000000 Vietnamese Dong-VND (~ £300–400) were associated with higher utility scores (*p* < 0.05 compared with completion up to secondary school and income of < 3000000 VND ~ £100, respectively) but not for EQ-VAS scores.

## Discussion

### Health profile across five health dimensions

Pain/discomfort and anxiety/depression were the dimensions of HRQoL where BC patients/survivors were more likely to report problems (75.4 and 57.3%, respectively)—these problems were 2–2.5 times more prevalent than any problems on other dimensions. In fact, these were also the two dimensions where severe or extreme problems were most likely to be reported by BC patients and survivors. The dimension where problems were least likely to be reported by those with BC was self-care (17.5%). These results are similar to findings from other research such as studies in China (*n* = 2626 BC patients), Korea (*n* = 827 BC patients), and Malaysia (*n* = 150 BC survivors) [19, 20, 25].

Pain/discomfort presents even years after the diagnosis and treatment as BC patients and survivors reported no difference in the level of problems in this dimension (79.2 and 73.5%, respectively). It also indicates that self-reported pain may not decrease for BC survivors after finishing treatment or may recur at given time points. In all other dimensions (mobility, self-care, usual activities, and anxiety/depression), the survivors had significantly better health status though the extent of the difference varied. The dimension with least difference between patients and survivors was usual activities (8%). The biggest difference between patients and survivors with respect to having problems lay on the dimensions of self-care and anxiety/depression (18%).

Compared to age-matched peers in the general population, BC patients and survivors were significantly more likely to have problems in every dimension, especially in relation to anxiety/depression. The difference between patients and general population in this dimension was approximately 46 percentage points whilst the difference between survivors and general population was 27 percentage points. Although the differences are larger than a similar study in Malaysia (compared survivors and general population), the trend is the same [25]. These results indicate the potentially profound psychological impact of BC on those who experienced the disease and imply the importance of not only care for physical health but also mental health of BC patients/survivors.

### EQ-VAS scores and utility scores

The mean (SD) EQ-VAS and utility scores of BC patients were 64.9 (20.1) and 0.74 (0.22) which were significantly lower than that of BC survivors at 76.2 (15.7) and 0.84 (0.15), respectively. EQ-VAS and utility scores of both BC patients and survivors were also lower than that of age-matched women from the general population at 77.9 (15.0) and 0.91 (0.1), respectively, which speaks to the face validity of the findings. The pattern is clear and consistent as patients have the lowest HRQoL and even when they survived and recovered from cancer, their HRQoL is still lower than age-matched women drawn from the general population. Both cross-sectional studies with matched women from general population [25, 26] and longitudinal study (using other measurement) [27] reported the same pattern of HRQoL. Compared with similar studies that used EQ-5D-5L, mean utility scores of BC patients in our study is quite similar with China and Malaysia but much lower than Korea (0.75 vs 0.78, 0.71, and 0.92, respectively) [19, 20, 25].

Apart from status of treatment (patients vs survivors), those who are younger, have higher education and household monthly income are more likely to report higher HRQoL. These results are similar to findings from previous studies, including studies that used different instruments for measurement [27–31] and are again suggestive of face validity. Other sociodemographic characteristics such as marital status and living residence area (urban/rural) are not significantly related to either EQ-VAS or utility scores.

Among BC patients, the observed trend in HRQoL amongst different stage of treatment was clear and consistent for both EQ-VAS and utility scores. Lowest HRQoL were reported by patients who have just gone through mastectomy (EQ-VAS = 54, utility score = 0.68). Patients who were being treated with chemotherapy, radiotherapy, or targeted therapy reported the next three lowest HRQoL (respectively, EQ-VAS = 61.8, 70.1, and 69.6; utility score = 0.71, 0.77, and 0.80). Patients who had just completed lumpectomy or breast reconstruction surgery reported better HRQoL than the survivors and on par or even higher than general population (in order of lumpectomy, breast reconstruction surgery, survivors, general population: 90.5, 80.0, 76.2, 77.9 for EQ-VAS; 0.88, 0.92, 0.84, 0.91 for utility score). The results were similar with a recent qualitative study in which Vietnamese BC patients claimed that mastectomy and chemotherapy were the two biggest challenges during their treatment (manuscript under minor revision). Participants in this study whilst expressing the hope to regain their body image with breast reconstruction surgery also shed light on the impact of mastectomy. One said “*I only know how bad it is after having a mastectomy… I feel so terrible. To be honest, whenever my husband holds me, I feel the emptiness… I hope that I can have reconstructive surgery because I cannot bear the defective body like that. I want my body to be normal again… Even if I die, I still want to die with a normal body… It is worse than losing my arm. Breast is a special symbol of women's beauty and every woman wants to be beautiful*”. Although the results were not statistically significant due to small sample size, it is consistent with previous findings of original studies and systematic reviews with meta-analysis [29, 30, 32–35].

### Strengths and limitations

This is the first study to report EQ-5D-5L data for BC patients and survivors in Vietnam. The use of the standardised EQ-5D-5L instrument also enabled the first comparative study of HRQoL of BC patients, survivors, and age-matched women from the country’s general population. It is important to note that the study was not informed by a formal power calculation though the sample size is comparable to similar studies in Vietnam and elsewhere [10]. Despite the small sample size, the study is able to provide novel and valuable insights into the HRQoL of BC patients and survivors in Vietnam, including its determinants as well as comparison with the general population. The results should be treated with some caution however as they may not generalize to the whole country as the study could not conduct a hospital-based survey in the South region (due to limited resources) which led to the under-representation of this region in the sample. In addition, it is important to note that our sample was dominated by respondents who lived in urban areas, were more educated, and had higher household monthly income. Those mostly came from the online survey (See Supplementary file 3 for sociodemographic characteristics of samples from online and hospital-based surveys). Thus, we acknowledge that there may exist differences between those who completed the online survey compared to those who completed the hospital-based survey related to education, rurality, and age that could impact the reported HRQoL.

## Conclusions

BC survivors showed higher HRQoL in various dimensions compared to patients who were receiving treatment but still much lower HRQoL than age-matched women in the general population. That anxiety/depression was much lowered among BC patients/survivors suggests more attention may be required with respect to their unmet psychological needs. Sociodemographic characteristics that appear to be independently associated with HRQoL include age (negative impact), education level (positive impact), and household monthly income (positive impact). These results should help inform future assessments of the comparative value for money of interventions intended to impact on BC in Vietnam.

## Supplementary Information

Below is the link to the electronic supplementary material.

Supplementary file1 (PDF 282 kb)

Supplementary file2 (PDF 282 kb)

Supplementary file3 (PDF 311 kb)

## Abbreviations

AIC: Akaike information criteria
BC: Breast cancer
BIC: Bayesian information criteria
EQ-5D-5L: EuroQol-5 dimensions-5 levels
GLM: Generalized linear models
HRQoL: Health-related quality of life
NA: Not applicable/available
NS: Not significant
OLS: Ordinary least square
SD: Standard deviation
VAS: Visual analogue scale
VND: Vietnamese dong

## Footnotes

1 Raw data were provided by the author of the original study [16].

## Declarations

### Data availability

All data generated or analysed during this study are included in this published article and supplementary materials.
