# Supplementary material for: Health-related quality of life among breast cancer patients compared to cancer survivors and age-matched women in the general population in Vietnam
Source: Qual Life Res. 2021 Sep 20;31(3):777–87. doi: 10.1007/s11136-021-02997-w (PMC8921138; doi:10.1007/s11136-021-02997-w)
Supplement: Supplementary file 1 — Supplementary file1 (PDF 282 kb) [file 11136_2021_2997_MOESM1_ESM.pdf]

The calculation of utility scores

In order to convert from EQ-5D-5L health profile to health utility score (or EQ-5D index value), the value set for Vietnam was used. This value set was obtained from an EQ-5D standardised valuation study conducted in 2017 with a representative sample of Vietnamese general population.[1] The full value set includes the index values (weight) of all 3125 (5^5) possible EQ-5D-5L health profiles. The following table presents a simplified version of the value set with the mean coefficients of regression models when moved from one level of problem to the next level in each dimension. These coefficients reflect the relative weight Vietnamese general population placed on different levels of problem in each of the five health dimensions.

**Health dimension**	**Level of severity**				
	No problems ①	Slight problems ②	Moderate problems ③	Severe problems ④	Extreme problems ⑤
Mobility	0	0.069	0.079	0.206	0.376
Self-care	0	0.043	0.046	0.147	0.231
Usual activities	0	0.046	0.059	0.173	0.299
Pain/Discomfort	0	0.084	0.152	0.270	0.367
Anxiety/Depression	0	0.064	0.113	0.171	0.239
Example in calculating utility score:<br>Value for health state 21235 = 1 – 0.069 – 0 – 0.046 – 0.152 – 0.239 = 0.494					

Reproduced with permission from Mai VQ (2020)[1]

Utility scores for heath profiles were calculated by subtracting the corresponding coefficients of the five dimensions from 1 (the value of full health or health state 11111). For example, the utility score of health profile 21235 = 1 – coefficient of slight problems in mobility – coefficient of no problems in self-care – coefficient of slight problems in usual activities – coefficient of moderate problems in pain/discomfort – coefficient of severe problems in anxiety/depression = 1 – 0.069 – 0 – 0.046 – 0.152 – 0.239 = 0.494. The subtracting represents the utility lost from being in a state of incomplete health.

[1] Mai VQ, Sun S, Minh HV, et al. An EQ-5D-5L Value Set for Vietnam. *Quality of Life Research* 2020.
