# Supplementary material for: Health-related quality of life among breast cancer patients compared to cancer survivors and age-matched women in the general population in Vietnam
Source: Qual Life Res. 2021 Sep 20;31(3):777–87. doi: 10.1007/s11136-021-02997-w (PMC8921138; doi:10.1007/s11136-021-02997-w)
Supplement: Supplementary file 2 — Supplementary file2 (PDF 282 kb) [file 11136_2021_2997_MOESM2_ESM.pdf]

Results from ordinary least square (OLS) regression models in relation to the assessment of the determinants of EQ-VAS and utility scores

Variable	EQ-VAS score				Utility score			
	Model 1[a] n=287		Model 2[b] n=306		Model 1[a] n=288		Model 2[b] n=296	
	β coeff	95% CI	β coeff	95% CI	β coeff	95% CI	β coeff	95% CI
**Treatment status**								
Patient[ref]								
Survivor	**9.3***	**4.4 – 14.2**	**9.0***	**4.3 – 13.6**	**0.06***	**0.01 – 0.11**	**0.07***	**0.02 – 0.11**
**Age group, years**								
<40[ref]								
40-49	-0.1	-5.2 – 5.0	-0.6	-5.4 – 4.2	-0.05	-0.09 – 0.00	-0.04	-0.09 – 0.00
50-59	-4.5	-10.9 – 1.8	-4.8	-10.6 – 1.0	-0.04	-0.10 – 0.02	-0.04	-0.09 – 0.02
60+	**-9.4***	**-17.1 – -1.6**	**-9.2***	**-16.3 – -2.1**	**-0.11***	**-0.18 – -0.03**	**-0.10***	**-0.17 – -0.03**
**Residence**								
Rural[ref]								
Urban	1.2	-4.3 – 6.6			0.03	-0.02 – 0.09		
**Education level**								
Completed up to secondary school[ref]								
Completed high school	4.7	-3.5 – 12.8	5.8	-1.2 – 12.9	0.05	-0.03 – 0.14	0.06	-0.02 – 0.15
Completed graduate	5.8	-1.3 – 12.9	**7.8***	**2.1 – 13.5**	0.06	-0.01 – 0.13	**0.08***	**0.01 – 0.15**
Completed postgraduate	7.4	-4.0 – 18.9	**9.6***	**0.4 – 18.8**	0.10	-0.00 – 0.19	**0.12***	**0.02 – 0.21**
**Marital status**								
Single/separated/divorce/widow[ref]								
Married	-1.3	-6.9 – 4.2			0.1	-0.01 – 0.07		
**Household monthly income**								
≤ 3,000,000 VND (~£100)[ref]								
3,000,001 – 6,000,000 VND (~£100-200)	2.0	-6.7 – 10.6			0.04	-0.06 – 0.14	0.04	-0.06 – 0.14
6,000,001 – 9,000,000 VND (~£200-300)	3.8	-5.2 – 12.9			0.07	-0.03 – 0.17	0.07	-0.03 – 0.17
9,000,001 – 12,000,000 VND (~£300-400)	2.4	-6.4 – 11.2			0.09	-0.01 – 0.18	**0.09***	**0.00 – 0.18**
> 12,000,000 VND (~£400)	3.0	-5.8 – 11.7			0.08	-0.02 - 0.17	0.08	-0.01 – 0.17
**AIC**	**2461**		**2603**		**-186.5**		**-201.6**	
**BIC**	**2513**		**2632**		**-135.2**		**-157.3**	

[a] Model with all exploratory variables | [b] Model included only variables with significant coefficients (Backward elimination)
*β coeff: Beta coefficients of the Tobit model | [ref]: Reference group | * p < 0.05 versus reference group*
VND: Vietnamese Dong. Exchange rate in October 2020: £1 ~ 30,000 VND

Results from generalized linear models (GLM) in relation to the assessment of the determinants of EQ-VAS and utility scores

Variable	EQ-VAS score				Utility score			
	Model 1[a †] n=287		Model 2[b †] n=306		Model 1[a §] n=288		Model 2[b §] n=296	
	β coeff	95% CI	β coeff	95% CI	β coeff	95% CI	β coeff	95% CI
**Treatment status**								
Patient[ref]								
Survivor	**9.3***	**4.8 – 13.8**	**9.0***	**4.8 – 13.2**	**0.08***	**0.02 – 0.14**	**0.08***	**0.02 – 0.14**
**Age group, years**								
<40[ref]								
40-49	-0.1	-5.7 – 5.5	-0.6	-5.7 – 4.6	-0.05	-0.10 – 0.00	-0.05	-0.10 – 0.00
50-59	-4.5	-10.7 – 1.6	-4.8	-10.5 – 0.9	-0.05	-0.11 – 0.02	-0.04	-0.11 – 0.02
60+	**-9.4***	**-16.3 – -2.4**	**-9.2***	**-15.6 – -2.8**	**-0.13***	**-0.22 – -0.03**	**-0.12***	**-0.21 – -0.04**
**Residence**								
Rural[ref]								
Urban	1.2	-4.1 – 6.4			0.03	-0.03 – 0.10		
**Education level**								
Completed up to secondary school[ref]								
Completed high school	4.7	-2.3 – 11.6	5.8	-0.2 – 11.9	0.08	-0.03 – 0.19	0.09	-0.12 – 0.20
Completed graduate	5.8	-1.1 – 12.7	**7.8***	**2.7 – 13.0**	0.09	-0.04 – 0.22	**0.11***	**0.02 – 0.20**
Completed postgraduate	7.4	-3.3 – 18.1	**9.6***	**0.8 – 18.4**	**0.13***	**0.01 – 0.25**	**0.15***	**0.04 – 0.26**
**Marital status**								
Single/separated/divorce/widow[ref]								
Married	-1.3	-6.7 – 4.0			0.03	-0.03 – 0.09		
**Household monthly income**								
≤ 3,000,000 VND (~£100)[ref]								
3,000,001 – 6,000,000 VND (~£100-200)	2.0	-5.4 – 9.3			0.06	-0.08 – 0.19	0.06	-0.07 – 0.19
6,000,001 – 9,000,000 VND (~£200-300)	3.8	-5.1 – 12.8			0.09	-0.04 – 0.22	0.09	-0.03 – 0.22
9,000,001 – 12,000,000 VND (~£300-400)	2.4	-5.5 – 10.4			0.11	-0.01 – 0.23	0.11	-0.01 – 0.23
> 12,000,000 VND (~£400)	3.0	-4.9 – 10.9			0.09	-0.03 - 0.21	0.10	-0.02 – 0.22
**AIC**	**2461**		**2602**		**-184.0**		**-199.9**	
**BIC**	**2513**		**2632**		**-132.7**		**-155.6**	

[a] Model with all exploratory variables | [b] Model included only variables with significant coefficients (Backward elimination)
† Family: Gaussian, Link: identity | § Family: Gaussian, Link: log
*β coeff: Beta coefficients of the Tobit model | [ref]: Reference group | * $p < 0.05$ versus reference group*
VND: Vietnamese Dong. Exchange rate in October 2020: £1 ~ 30,000 VND
