# Supplementary material for: Health-related quality of life among breast cancer patients compared to cancer survivors and age-matched women in the general population in Vietnam
Source: Qual Life Res. 2021 Sep 20;31(3):777–87. doi: 10.1007/s11136-021-02997-w (PMC8921138; doi:10.1007/s11136-021-02997-w)
Supplement: Supplementary file 3 — Supplementary file3 (PDF 311 kb) [file 11136_2021_2997_MOESM3_ESM.pdf]

Table S1 Sample characteristics, compared between online survey and hospital-based survey

Characteristics	Online survey n (%)	Hospital-based survey n (%)	p-value*	Total n (%)	National data %
**Total**	209 (67.6)	100 (32.4)		309 (100.0)	
**Age, mean (range)**	46 (28-69)	52 (31-86)	<0.001	48 (28-86)	NA
**Residence area: urban**	173 (86.1)	38 (38.0)	<0.001	211 (70.1)	35.0[b]
**High education level (completed at least high school)**	199 (96.1)	38 (38.0)	<0.001	237 (77.2)	20.8[a]
**Occupation (employee or wage worker)**	115 (56.1)	18 (18.0)	<0.05	133 (43.6)	45.0[c]
**Household monthly income**					
≤ 3,000,000 VND (~£100)	13 (6.6)	30 (30.3)	<0.001	43 (14.5)	NA
3,000,001 – 6,000,000 VND (~£100-200)	31 (15.7)	26 (26.2)		57 (19.9)	
6,000,001 – 9,000,000 VND (~£200-300)	19 (9.6)	12 (12.1)		31 (10.4)	
9,000,001 – 12,000,000 VND (~£300-400)	55 (27.8)	20 (20.2)		75 (25.3)	
> 12,000,000 VND (~£400)	80 (40.4)	11 (11.1)		91 (30.6)	
**Completion status of treatment**					
No (Patient)	45 (21.5)	55 (55.0)	<0.001	100 (32.4)	NA
Yes (Survivor)	164 (78.5)	45 (45.0)		209 (67.6)	
**Stage of breast cancer at diagnosis:** Late stage (Stage III and IV)	28 (13.4)	15 (15.0)	NS	43 (13.9)	49.5[d]

** Results of Chi-square tests compared between two administrative types of the survey*
[a] Data are from Vietnam Population and Housing Census 2009[1]
[b] Data are from Statistical Summary Book of Vietnam 2017[2]
[c] Data are from 2018 report of the Ministry of Labour – Invalids and Social Affairs[3]
[d] Data from the 5 biggest oncology hospitals in Vietnam (2009)[4]
NA: Data not available/not applicable | NS: not significant
VND: Vietnamese Dong. Exchange rate in October 2020: £1 ~ 30,000 VND

[1] Vietnam General Statistics Office, Central Population and Housing Census Steering Committee. Vietnam Population and Housing Census 2009. Hanoi, Vietnam; 2009.
[2] General Statistics Office of Vietnam. Statistical summary book of Vietnam 2017. Hanoi, Vietnam: Statistical Publishing House; 2017. 424 p.
[3] Ministry of Labour – Invalids and Social Affairs. Decision No. 1052/QĐ-LĐTBXH: Annoucement of the results of 2018 poor and near-poor household survey. Hanoi, Vietnam. 2019.
[4] Thuan TV, Anh PT, Tu DV, Huong TTT. Cancer control in Vietnam: Where are we. Cancer care in emerging health systems [Internet]. 2016. Available from: http://www.cancercontrol.info/cc2016/cancer-control-in-vietnam-where-we-are/.
